# Diffusion in Copper/Cobalt Systems under High Magnetic Fields

**DOI:** 10.3390/ma14113104

**Published:** 2021-06-05

**Authors:** Zhiwei Zhang, Xiang Zhao, Sadahiro Tsurekawa

**Affiliations:** 1 Key Laboratory for Anisotropy and Texture of Materials (Ministry of Education), School of Materials Science and Engineering, Northeastern University, Shenyang 110819, China; zhiweizhang@stumail.neu.edu.cn; 2 Division of Materials Science and Chemistry, Faculty of Advanced Science and Technology, Kumamoto University, Kurokami 2-39-1, Kumamoto 860-8555, Japan

**Keywords:** high magnetic field, Cu/Co diffusion couple, atomic diffusion, frequency factor, electron probe micro analyzer

## Abstract

Comprehensive research on a high magnetic field’s effect on diffusion is lacking; hence, this study investigates the effect of the magnetization of such a field on diffusion using a copper/cobalt diffusion couple in the diamagnetic/ferromagnetic states, respectively. The diffusion couple was formed using explosive welding to avoid diffusion during manufacturing. The diffusion couple annealed within a temperature range of 1165–1265 K under a 0–6-T high magnetic field. The angle between the diffusion and magnetic field directions was set as 0° and then 180°. The penetration profiles of cobalt volume diffusion in the copper and grain-boundary diffusion of copper in cobalt were constructed using an electron probe micro analyzer. The high magnetic field increased the volume diffusivity of cobalt in copper, but had no evident effect on the grain-boundary diffusivity of copper in cobalt, irrespective of the magnetic field direction. An Arrhenius plot of the cobalt volume diffusivity in copper demonstrated that the applied high magnetic field enhanced diffusion by changing the frequency factor rather than the activation energy; this can be attributed to the increased diffusion entropy caused by changing the vacancy concentration, which resulted from the introduction of magnetization under a high magnetic field.

## 1. Introduction

Extensive studies have demonstrated that applying a high magnetic field during heat treatment is an effective control method for modifying material microstructure because it affects several metallurgical phenomena such as grain boundary migration [1], texture formation [2], segregation [3], and ordering [4]. Therefore, a material’s microstructure design can be manipulated, and consequently, its material properties can be enhanced by applying a high magnetic field. Microstructure development is closely related with atomic diffusion. Thus, exploring the effects of a high magnetic field on atomic diffusion is the precondition and foundation of microstructure design.

Recently, research on this subject has been focused on the effect of high magnetic fields on the atomic diffusion of diffusion couples in the paramagnetic/paramagnetic states (Al/Mg [5,6], Ni/Al [7], Ti/Ni [8], and Ti/Fe [9]) and paramagnetic/diamagnetic states (Al/Al–3% Cu [10], Cu/Ni [11,12], Fe/Fe–50 wt.% Si [13], Fe/Fe–20 wt.% Si [14]). Several studies [5,7,10,11,13] have suggested that a high magnetic field restrains atomic diffusion when the diffusion direction is perpendicular to the magnetic field. Other studies [6,12,14] have revealed that a high magnetic field parallel to the diffusion direction promotes the diffusion process. However, some studies [8,9] found no evident effect of high magnetic fields on the atomic diffusion in the case of titanium. Although a high magnetic field has different effects on material diffusivities under different diffusion heat treatment conditions, it can be concluded that high magnetic fields modify the frequency factor rather than the activation energy in all atomic diffusion processes.

However, few studies have investigated the effect of high magnetic fields on the atomic diffusion of diffusion couples in ferromagnetic/paramagnetic and ferromagnetic/diamagnetic states. Pokoev and Fedotov [15] studied a diffusion system under ferromagnetic and paramagnetic conditions and examined the effect exerted by a uniform magnetic field on the 63Ni diffusion in polycrystalline Armco-Fe, single crystalline silicon Fe, and polycrystalline Co (Bologna, Italy). They reported that the temperature dependence of 63Ni volume diffusivities in the ferromagnetic state under a uniform magnetic field is of a non-Arrhenius character. Pokoev [16] also reported the non-monotonous nature of the dependence of 63Ni diffusivities on the magnetic field during grain-boundary diffusion in α-Fe under a uniform magnetic field. Fujii et al. [17] studied the diffusion system under the ferromagnetic and diamagnetic states, investigating the carbon diffusion in iron under a high magnetic field using an Fe/Fe–0.87 wt.% C diffusion couple. They concluded that a uniform magnetic field reduces the carbon diffusion coefficient in α-Fe. Moreover, Wang [18] found that the application of a high magnetic field reduced the Kirkendall effect in Cu/Co diffusion couple within the temperature range of 1243–1303 K. However, the results were not confirmed because the heat treatment temperature was relatively high, which could reduce the effect of the high magnetic field. In addition, an Arrhenius plot could not be found under the conditions of applying or not applying a high magnetic field. Therefore, the nature of diffusion in the ferromagnetic state under high magnetic fields still require further research.

In this research, the diffusion behavior under the magnetism of a high magnetic field was studied by choosing Cu/Co as a diffusion couple. The twofold reasons why Cu/Co system was selected were as follows. One was that the cobalt has a relatively high Curie temperature. That made sure cobalt was in a ferromagnetic state during annealing. The other was that no intermediate phases were formed during annealing. Thus, the nature of atomic diffusion could be clearly revealed. With the Cu/Co diffusion couple, the volume diffusivity of cobalt in copper and the grain-boundary diffusivity of copper in cobalt under a high magnetic field were estimated. The results of this work clarify the impact of the intensity and the direction of high magnetic field on the diffusion behaviors in a system containing a ferromagnetic state, are expected to lay a theoretical foundation for the microstructure design of ferromagnetic materials under high magnetic fields.

## 2. Experimental

### 2.1. Preparation of Diffusion Couples

**Figure 1:**
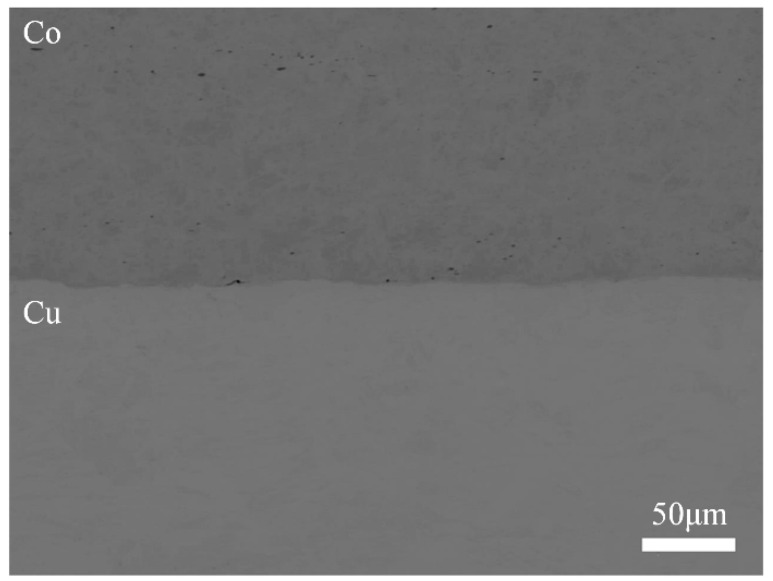
Cross-section of the diffusion couple before annealing.

The diffusion behavior in a copper–cobalt system under a high magnetic field was investigated through a diffusion couple of pure copper (99.96 wt.%) and pure cobalt (99.9 wt.%). The pure copper and pure cobalt plates were combined to form diffusion couples using an explosive welding technique. This experiment was conducted at the Shock Wave and Condensed Matter Research Center of Kumamoto University. An ammonium nitrate-fuel oil (ANFO) mixture-based explosive (ANFO-A) was employed in this welding process. Before welding, a 5-mm-thick copper plate and a 1-mm-thick cobalt plate were annealed at 681 K for 21.6 ks and 1238 K for 10.8 ks, respectively, under vacuum. Next, they were mechanically ground to obtain a clean surface. Immediately after welding, 6 mm × 5 mm × 1 mm specimens were cut from the welded copper and cobalt clad. The cross sections of the specimens were polished using waterproof papers (#400–#4000) and oil-based diamond slurry (1 μm and 0.25 μm). Figure 1 shows the interface of the diffusion couples before diffusion annealing, which indicates a good binding between the copper and cobalt plates.

### 2.2. Diffusion Annealing under a Static Magnetic Field

The experimental device consisted of a superconducting magnet with a maximum field intensity of 6 T and an electric furnace equipped with a Mo sheet heater with a maximum heating temperature of 1773 K. The magnet (HF6-100VHT-1, bore diameter = 100 mm) was supplied by Sumitomo Heavy Industries, Ltd (Tokyo, Japan). Tungsten sheets were placed between the specimen and a specially designed carbon holder to prevent carbon contamination. The bonding interface of the couples was placed in the center of the high magnetic field in the perpendicular direction to that of the magnetic field. The angle between the cobalt diffusion direction (DD) and magnetic field direction (MFD) were set as 0° or 180° to investigate the effect of the magnetic field direction on atom diffusivity. Diffusion annealing was carried out from 1165 K to 1265 K with and without a high magnetic field under vacuum (1.0 × 10^−3^ Pa). The Curie temperature of cobalt is 1394 K, which ensures that the cobalt is in a ferromagnetic state during the diffusion annealing. Based on the results of previous studies, the annealing time was chosen to ensure the cobalt volume diffusion depth was not less than 80 μm [19,20,21]. Table 1 lists the detailed parameters of the magnetic field annealing process.

### 2.3. Evaluation of Atom Diffusivity

The atom concentration distribution dependence on the penetration depth was assessed by electron probe micro analyzer (EPMA, EPMA-1720H, Shimadzu, Kyoto, Japan) at an accelerating voltage of 15 kV and with a minimum beam size was used to ensure the accuracy of the measurement. The cobalt volume concentration in copper was measured beginning from the interface by point analysis in the grain far from the grain boundary. The cobalt volume diffusivity in copper was determined by Fick’s second law. The grain-boundary concentration of copper in cobalt was evaluated by the mapping analysis method using EPMA. Then, the grain-boundary diffusivity of copper in cobalt was calculated based on the integral representation of the exact solution derived by Whipple [22] from the Fisher model [23].

## 3. Results

### 3.1. Distribution of Cobalt and Copper in the Annealed Cu/Co Diffusion Couple with and without a High Magnetic Field

**Figure 2:**
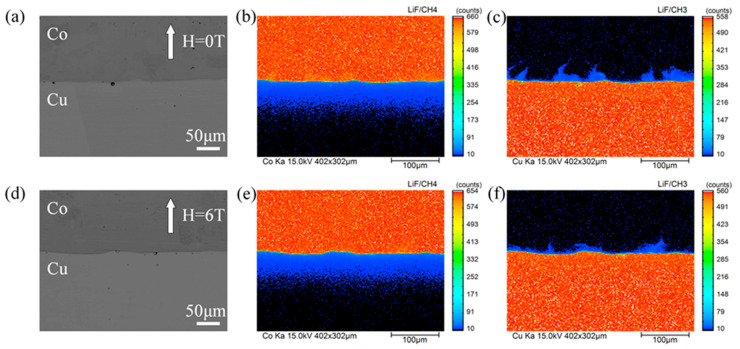
EPMA mapping analysis of the diffusion couple annealed at 1214 K for 9 h with and without the high magnetic field. The angle between the magnetic field direction and the cobalt diffusion direction is 180°. (**a**,**d**) back scattered electron (BSE) images for 0 T and 6 T, (**b**,**c**) corresponding cobalt distribution and copper distribution of (**a**), respectively, (**e**,**f**) corresponding cobalt distribution and copper distribution of (**d**), respectively.

The distribution of cobalt and copper were measured in the cross section of the Cu/Co diffusion couple after annealing with and without a high magnetic field. Figure 2 shows a typical back scattered electron (BSE) micrograph and EPMA mapping analysis of the diffusion couple annealed at 1214 K for 9 h under a nonmagnetic and a 6-T magnetic fields. In Figure 2a,d, no visible intermetallic layers were observed at the interface of the annealed diffusion couple. According to the Equilibrium phase diagram, this may be attributed to the fact that no intermetallic phase could exist at an annealing temperature range of 1165–1265 K [24]. The concentration gradient at the interface of the diffusion couple could not be detected in the BSE images because of the low mutual solubility of copper and cobalt in solid phases and the machine limitation of the Shimadzu EPMA-1720H. From the EPMA mapping analysis of cobalt distribution (Figure 2b,e) and the high annealing temperature (86% to 93% of the copper melting point), it can be determined that the type of cobalt diffusion in this work is volume diffusion classified as type A according to Harrison’s classification [25]. Figure 2c,f indicate that copper diffusion occurred along the grain boundary and leakage to the volume. As the annealing temperatures was 66% to 72% of the cobalt melting point, the diffusion behavior of copper in cobalt is considered a type B diffusion (based on Harrison’s classification). Further, the high magnetic field effects on the cobalt (ferromagnetic state) volume diffusivity in copper (diamagnetic state) and the copper (diamagnetic state) grain boundary diffusivity in cobalt (ferromagnetic state) are discussed in Section 3.2 and Section 3.3, respectively.

### 3.2. Cobalt Volume Diffusivity in Copper under a High Magnetic Field

**Figure 3:**
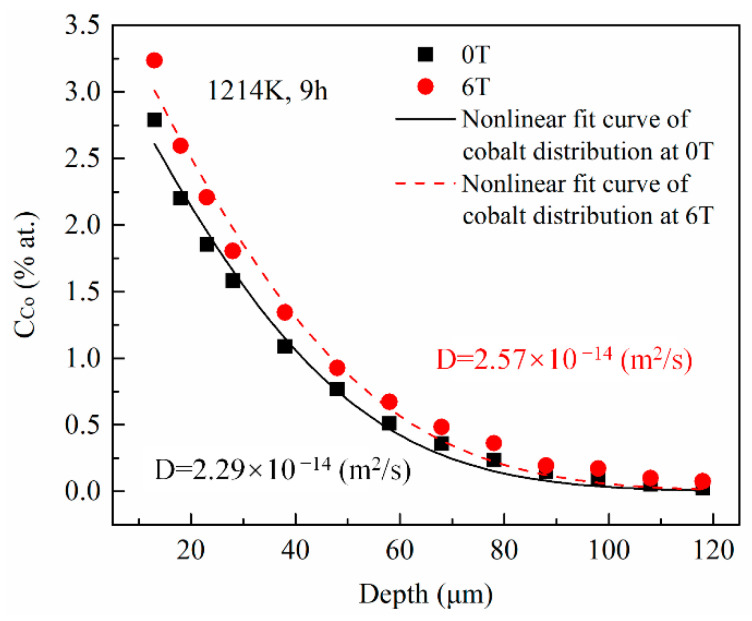
The cobalt concentration profiles in copper annealed at 1214 K with and without the high magnetic field. The diffusivities of cobalt in copper were estimated using the nonlinear curve fitting based on the error function (the solid and the dashed lines). The angle between the magnetic field direction and the cobalt diffusion direction is 180°.

The cobalt diffusion coefficients in copper with and without a high magnetic field were calculated using a fitting procedure. For each annealing condition, 6 positions were investigated. Figure 3 illustrates the typical concentration dependence on the penetration depth for specimens annealed at 1214 K under a nonmagnetic and a 6-T magnetic fields, respectively, with the angle between the MFD and DD = 180°. The cobalt concentration in copper exponentially decreased with increasing the penetration depth with and without the high magnetic field. Nonlinear fitted curves (the solid line for 0T and the dashed line for 6T in Figure 3) are obtained based on the error function distribution of cobalt in copper discussed in the following paragraph. Then, the cobalt volume diffusion coefficients were calculated at each position under each annealing condition. The correlation coefficients of the fitting procedure were higher than 0.98 (93.8% of them were higher than 0.99). The maximum and minimum results under each annealing condition were removed and the average value of the remaining 4 results were used as the final cobalt volume diffusion coefficients value. The results imply that the volume diffusivity of cobalt in copper was increased by 6%–21% under a 6-T magnetic field.

The case here is a typical semi-infinite diffusion couple of two dissimilar materials with no effect of component diffusion on the edges of the materials. The solution in this case is demonstrated by Equation (1).

(1) $$C_{Co}\left(x,t\right)=C_{0}\left[1-erf\left(\frac{x}{2\sqrt{Dt}}\right)\right]$$

**Figure 4:**
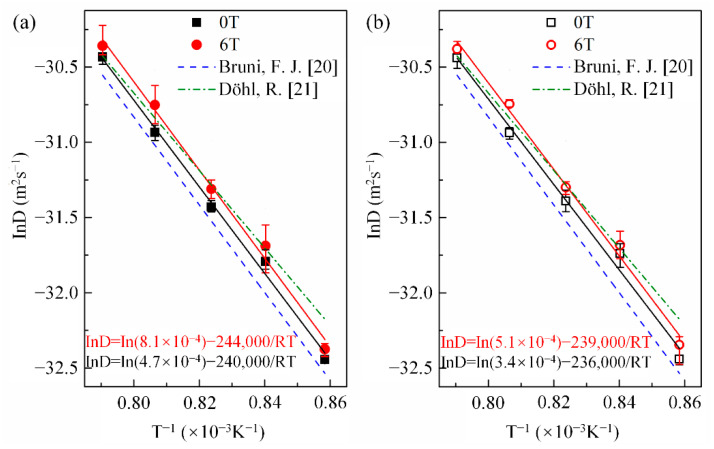
Arrhenius plots for cobalt diffusivity in copper with and without a high magnetic field. The angle between the magnetic field direction and the cobalt diffusion direction: (**a**) 0° and (**b**) 180°. For comparison, the previous data of Bruni, F. J. [20] and Döhl, R. [21], which were measured under the nonmagnetic field, are presented by the dash and dash-dot lines, respectively.

**Figure 5:**
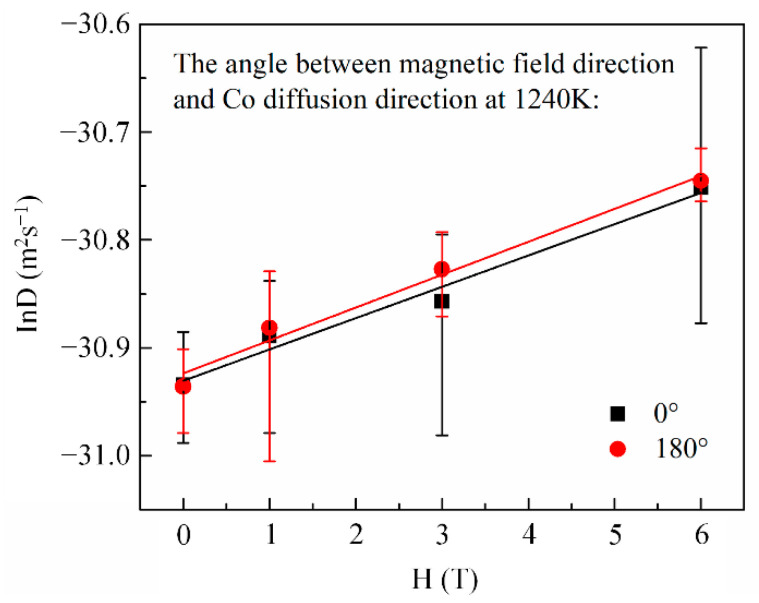
The linear dependence of cobalt diffusion coefficient in copper on magnetic field intensity.

Here, x is the penetration depth of cobalt in copper, $C_{0}$ is the cobalt concentration in copper at the initial contact plane (x=0), *t* is the diffusion annealing time, erf is the error function, and *D* is the cobalt diffusion coefficient. Following Equation (1), the cobalt volume diffusivities were estimated from the nonlinear curve fitting method illustrated in Figure 3. Figure 4 shows the cobalt diffusion coefficients in copper calculated under each annealing condition. For comparison, the literature data for cobalt volume diffusivities in copper [20,21] measured without a high magnetic field are plotted in dashed lines. Figure 4 indicates that there is an approximate linear Arrhenius relationship in this temperature range, irrespective of the existence and the direction of the magnetic field. The frequency factor and activation energy of diffusion were evaluated using the intercept and slope of the Arrhenius plots fitted by the least square method, respectively. All the correlation coefficients of the linear fitting were higher than 0.99. The results are listed in Table 2. It is concluded that a high magnetic field could significantly increase the cobalt volume diffusivities in copper by affecting the frequency factor $D_{0}$ rather than the activation energy *Q*, irrespective of the angle between DD and MFD (0° or 180°). Figure 5 illustrates the dependence of cobalt atom diffusivities in copper on the magnetic field intensity at 1240 K and with the angle between DD and MFD = 0° or 180°. The natural logarithm of the cobalt diffusivity increased linearly with the increase in the magnetic field intensity.

### 3.3. Grain-Boundary Diffusivity of Copper in Cobalt under a High Magnetic Field

The grain-boundary diffusion coefficients of copper in cobalt with and without a high magnetic field were estimated using Whipple’s exact analytical solution derived from the Fisher model. Under each annealing condition, two areas (402 μm × 302 μm) were investigated. To calculate the average copper concentration based on the copper penetration depth, the interface was flattened using a method combining Python and MATLAB. The values of the triple product $P=s\delta D_{b}$ under each annealing condition were calculated based on Whipple’s solution [22] of a constant source with two conditions ($\alpha^{*}<0.1$ and $\beta^{*}>10$).

(2) $$s\delta D_{b}=1.322{\left(D/t\right)}^{1/2}{\left(-\partial ln\bar{c}/\partial y^{6/5}\right)}^{-5/3}$$

(3) α∗=sδ2(Dt)12

(4) β∗=sδDb2D(Dt)12

where $\bar{c}$ is the average concentration of every penetration depth, *y* is the penetration depth, *s* is the segregation factor, *δ* is the average grain boundary width, *t* is the diffusion annealing time, *D* is the volume diffusivities of copper in cobalt, and $D_{b}$ is the grain boundary diffusivities of copper in cobalt. In this study, the volume diffusivities of copper in cobalt at $D_{0}$ ≈ 1×10^−4^ m^2^/s and *Q* = 275 kJ/mol [26] were used. Thus, the triple product $P=s\delta D_{b}$ of the grain-boundary diffusion of copper under each annealing condition could be calculated.

**Figure 6:**
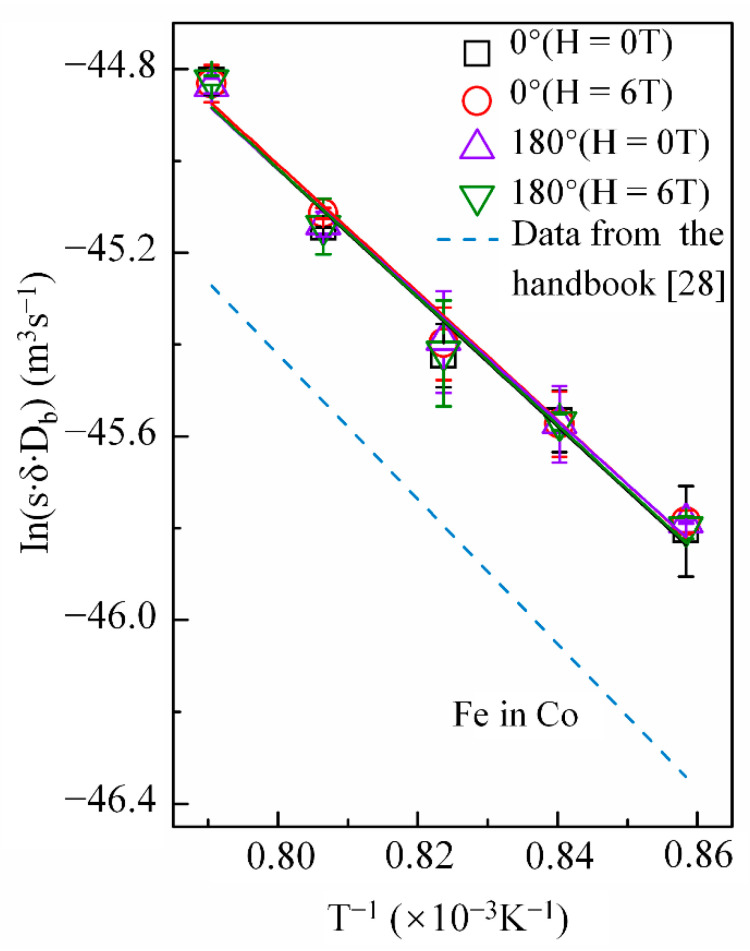
Arrhenius plots for grain boundary diffusivity of copper in cobalt with and without a high magnetic field. The angle between the magnetic field direction and the cobalt diffusion direction is 0° or 180°. The same type of the line with respect to the grain boundary diffusion data of iron in cobalt [28] was drawn by the dashed line.

Cobalt volume diffusion in copper is governed by a vacancy mechanism, while grain-boundary diffusion of copper in cobalt occurs through several mechanisms [27]. Therefore, a high magnetic field may affect the diffusion behaviors of cobalt in copper and copper in cobalt differently. Figure 6 shows the temperature dependence of *p* in the Arrhenius form under a nonmagnetic field and a 6-T magnetic field with the angle between the copper DD and MFD = 0° or 180°. As mentioned, the high magnetic field enhanced the cobalt volume diffusion in copper, irrespective of the angle between DD and MFD. However, it did not exhibit any effect on the grain-boundary diffusivities of copper (Figure 6). The *p* value obtained in this experiment was reasonable compared to that of iron in cobalt retrieved from the literature [28] (Figure 6) and the energy of the grain-boundary self-diffusivities of cobalt (117.2 kJ/mol) [29]. Table 3 lists the frequency factor ${\left(s\delta D_{b}\right)}_{0}$ and activation energy *Q* of diffusion obtained by the intercept and slope of the linear fit of the Arrhenius plots, respectively.

## 4. Discussion

### 4.1. The Effect of a High Magnetic Field on the Cobalt Volume Diffusivity in Copper

Considering the vacancy mechanism, the volume diffusivity *D* is described by Equation (5) [30]:

(5) $$D=\frac{1}{n}a^{2}zve^{\left[\left(\Delta S_{vac}+\Delta S_{mig}\right)/R\right]}e^{\left[-\left(\Delta H_{vac}+\Delta H_{mig}\right)/RT\right]}=D_{0}e^{\left(-Q/RT\right)}$$

where *n* is a constant, *a* is the diffusion atomic-jump length, *z* is the coordination number, *v* is the atomic vibration frequency, *R* is the gas constant, and *T* is the absolute temperature. $D_{0}=\frac{1}{n}a^{2}zve^{\left[\left(\Delta S_{vac}+\Delta S_{mig}\right)/R\right]}$ and $Q=\Delta H_{vac}+\Delta H_{mig}$, where $D_{0}$ is the frequency factor of diffusion; *Q* is the activation energy; $\Delta S_{vac}$ and $\Delta H_{vac}$ are the entropy and enthalpy of vacancy formation, respectively; and $\Delta S_{mig}$ and $\Delta H_{mig}$ are the entropy and enthalpy of migration, respectively. The experimental results statistically indicate that a high magnetic field could affect the frequency factor, $D_{0}$, rather than the activation energy, *Q*, during the diffusion process, irrespective of the angle between DD and MFD (0° or 180°). DD was parallel to MFD, and no Lorentz force could generate between them regardless of the angle between them. Additionally, a uniform magnetization was obtained in specimens subjected to a static magnetic field. In addition, this result is consistent with the finding reported by Pokoev [31]. Therefore, the angle between DD and MFD (whether 0° or 180°) does not affect the experimental results. A similar conclusion to the one obtained here was obtained by studies that investigated the effect of a high magnetic field on the diffusion process [5,6,7,10,11,12,13,14]. They suggested that atomic diffusivity is affected by the frequency factor rather than the activation energy. The vibration frequency, v, of the atoms and the diffusion atomic-jump length, a, would not significantly change under a high magnetic field [13,17], and hence, the increase in the cobalt diffusivity is mainly associated with the increase in the diffusion entropy under a high magnetic field. In addition, the natural logarithm of the cobalt diffusivity increased linearly with the increase in the magnetic field intensity (Figure 5); thus, the diffusion entropy should have a linear relationship with the magnetic field intensity. Consequently, the increase in the cobalt volume diffusion coefficient in copper could be ascribed to the increase in the diffusion entropy induced by the magnetic field.

The role of the increase in the magnetic free energy of a diamagnetic copper material in enhancing the diffusion entropy of the cobalt diffusion process under a high magnetic field was also discussed. The magnetic free energy, U, per unit volume of a diamagnetic material is given by Equation (6).

(6) $$U=-\frac{1}{2}\mu_{0}\chi H^{2}$$

where $\mu_{0}$ is the vacuum magnetic permeability, χ is the susceptibility, and *H* is the magnetic field intensity. Because the value of the susceptibility, χ, of copper is negative, the magnetic free energy of the copper matrix is positive. Based on the first law of thermodynamics, the internal energy of the copper matrix could increase under a high magnetic field. Anand et al. [32] observed a suppression in the copper diffusivity in iron below the curie temperature and explained the phenomenon based on the theories of spin-ordering and elastic constants dependance on temperature. A larger decline in the vacancy concentration was observed in the ferromagnetic state compared to that in the paramagnetic state. Furthermore, in the paramagnetic region [9] or diamagnetic region, the magnetization induced by a magnetic field occurs under a high magnetic field. Similar to Anand et al., another study concluded that the change in the internal energy causes by a high magnetic field has a major effect on the vacancy concentration [9]. A new vacancy forming in copper (in the diamagnetic matrix) under a high magnetic field may lead to a reduction in the absolute value of susceptibility, which has structural sensitivity [33,34]. Thus, it causes a decline in the magnetic free energy of the unit volume. This suggests that the fraction of thermal vacancies at equilibrium, $X_{v}$, in the copper matrix during high temperature annealing under a high magnetic field might increase to enhance the thermodynamic stability of the matrix. The increase in the number of vacancies contributes to the increase in the vacancy formation entropy and configurational entropy [35]. The enhancement of vacancy formation entropy ($\Delta X_{v}\Delta S_{v}$, where $\Delta S_{v}$ is the change in the vacancy formation entropy for one mole of vacancy, and $\Delta X_{v}$ is the mole fraction of the vacancies introduced) can be attributed to irregular vibrations of atoms surrounding the introduced vacancies. The increase in the configurational entropy can be attributed to the increase in the possible arrangements between the components and vacancies. The relationship between the configurational entropy, $\Delta S_{Config}$, and the vacancy concentration, $X_{v}$, of a system is given by Equation (7).

(7) $$\Delta S_{Config}=-R\left[X_{v}lnX_{v}-\left(1+X_{v}\right)\ln\left(1+X_{v}\right)\right]$$

Thus, the configurational entropy increases with the increase in the vacancy concentration. In addition, when a vacancy is introduced, it causes a certain relaxation of a region involving several atoms [30]. When the configurational entropy increases by the increase in the introduced vacancy concentration, the total relaxation of the lattice increases. During the diffusion process dominated by the vacancy mechanism, the jumping atom has to move between neighboring atoms [30]. The neighboring atoms are more displaced at the saddle point than that during the process with lower configurational entropy. The migration entropy is the change in the lattice vibration that corresponds to the movement of the jumping atom from its equilibrium position to the saddle point [36]. Therefore, the migration entropy increases due to the enhancement in the configurational entropy in the presence of a high magnetic field. In conclusion, when a high magnetic field is applied, the vacancy formation and migration entropies increase due to the increase in the vacancy concentration in the copper matrix. Thus, the cobalt volume diffusion coefficient in copper increases due to the significant improvement in the diffusion entropy.

To summarize, high magnetic fields can affect the diffusion entropy through changing the vacancy concentration, thus affecting the cobalt volume diffusion process in copper, irrespective of the angle between DD and MFD (0° or 180°). The increase in the cobalt volume diffusivity in copper under a high magnetic field can be attributed to the increase in the diffusion entropy rather than the change in the activation energy. However, the effect of high magnetic fields on the vacancy concentration cannot be studied using the available technology. Thus, further work is still required using future technologies to experimentally confirm this explanation.

### 4.2. The Effect of a High Magnetic Field on the Grain-Boundary Diffusivity of Copper in Cobalt

In contrast to volume diffusion, grain-boundary diffusion typically occurs is through the collective jumps of atoms [27]. Generally, depending on the temperature, grain-boundary diffusion would be 4 to 6 orders of magnitude faster than volume diffusion [37]. According to the experimental results, the effect of a high magnetic field is comparable to the chemical potential gradient of cobalt on its volume diffusion, which is the real driving force for diffusion. Thus, the effect of the magnitude of the high magnetic field on the grain-boundary diffusion of copper is significantly less than that of the chemical potential gradient at the grain boundary. However, a study [16] reported a non-monotonic effect of magnetic fields on the volume and grain-boundary diffusions of 63Ni in α-Fe under a 0–0.7-T uniform magnetic field at ferromagnetic temperatures. Nevertheless, the average value of the grain-boundary diffusion coefficient of 63Ni obtained under different intensities of a uniform magnetic field is equal to the value obtained under a nonmagnetic field. In addition, the field-induced increase and decrease in the grain-boundary diffusivity of 63Ni were not significant compared to those in the volume diffusivity of 63Ni. Therefore, high magnetic fields do not have a pronounced effect on the grain-boundary diffusion of copper, irrespective of the angle between DD and MFD (0° or 180°).

## 5. Conclusions

The effect of a high magnetic field on the diffusion in a copper/cobalt system was experimentally investigated using a diffusion couple consisting of pure copper and pure cobalt. The main results are summarized as follows:

1. According to Harrison’s classification, the diffusion of cobalt in copper and that of copper in cobalt were considered type A and type B diffusions.
2. In contrast to the enhancement of the cobalt volume diffusion in copper induced by the high magnetic field, which is independent of the angle between DD and MFD (0° or 180°), no visible effect of the magnetic field on the grain-boundary diffusivities of copper in cobalt.
3. The high magnetic field can increase the cobalt volume diffusivity by increasing the frequency factor, whereas it has a lower effect on the activation energy.
4. This improvement is a result of the increase in diffusion entropy caused by the change in the vacancy concentration in the copper matrix under the high magnetic field.

## Figures and Tables

**Table 1:** Parameters of the magnetic field annealing for copper and cobalt diffusion couple.

Temperature *T*/K	Time *t*/h	The Angle between MFD and Co DD	Magnetic Field Intensity *H*/T
1265	4.5	0°/180°	0
1265	4.5	0°/180°	6
1240	6.6	0°/180°	0
1240	6.6	0°/180°	1
1240	6.6	0°/180°	3
1240	6.6	0°/180°	6
1214	9	0°/180°	0
1214	9	0°/180°	6
1190	12	0°/180°	0
1190	12	0°/180°	6
1165	15	0°/180°	0
1165	15	0°/180°	6

**Table 2:** Frequency factor $D_{0}$ and activation energy Q (standard deviation for 95% confidence) for cobalt volume diffusivities in copper under a nonmagnetic field and a 6-T magnetic field.

The Angle between MFD and DD of Co	Magnetic Field Intensity *H*/T	Frequency Factor *D*_0_/m^2^ s^−1^	Activation Energy *Q*/kJmol^−1^
0°	0	4.7 × 10^−4^	240 ± 30
0°	6	8.1 × 10^−4^	244 ± 36
180°	0	3.4 × 10^−4^	236 ± 41
180°	6	5.1 × 10^−4^	239 ± 35

**Table 3:** Frequency factor ${\left(s\delta D_{b}\right)}_{0}$ and activation energy *Q* (standard deviation for 95% confidence) for copper grain boundary diffusivities in cobalt under a nonmagnetic field and a 6-T magnetic field.

The Angle between MFD and DD of Cu	Magnetic Field Intensity *H*/T	Frequency Factor ${\left(s\delta D_{b}\right)}_{0}/m^{3}s^{-1}$	Activation Energy *Q*/kJmol^−1^
0°	0	2.1 × 10^−15^	116 ± 31
0°	6	2.0 × 10^−15^	116 ± 24
180°	0	1.7 × 10^−15^	114 ± 23
180°	6	2.0 × 10^−15^	116 ± 29

## Data Availability

Data sharing is not applicable to this article.
